# Supplementary material for: The older the injured, the worse the outcomes: A comparison of injury patterns and in-hospital outcomes between younger and older adult trauma patients at a tertiary hospital in Northern Tanzania
Source: PLOS Glob Public Health. 2025 Jun 2;5(6):e0004547. doi: 10.1371/journal.pgph.0004547 (PMC12129321; doi:10.1371/journal.pgph.0004547)
Supplement: Attachment — Submitted filename: Response to reviewers - ET Paper .docx [file pgph.0004547.s002.docx]

**To:**

**PLOS Global Public Health Editorial Board**

**RE: Response to reviewer comments for manuscript PGPH-D-24-02377**

Dear reviewers and editors,

Thank you for all the comments and concerns raised to improve this manuscript for publication at PLOS Global Public Health. We have responded to the comments as follows:

**Reviewer #1:**

1. In the research objectives, your study addresses an important issue of trauma care in Tanzania’s aging population. However, it could benefit from explicitly linking the findings to actionable healthcare policies or interventions, which is currently missing…

***Answer:***

***We have linked our findings to the adoption and implementation of clinical practice guidelines (CPGs)/Geriatric protocols for older adult trauma care in the conclusion section.***

2. In the methodology, using secondary data from the trauma registry is a good approach, but it lacks details about how missing or incomplete data were handled. Adding this would clarify potential biases and enhance transparency.

***Answer:***

***We reported to use a complete case analysis approach, and have added details why in the data analysis section. We also reported the number of missing observation for every variables with missingness.***

3. The statistical analysis is thorough and appropriate, especially with multivariable models for mortality. However, the manuscript doesn’t explain why certain statistical tests were chosen or how missing data were addressed, including these would help strengthen the analysis.

***Answer:***

***We have explained all the statistical test applied for the descriptive analysis and why, In this study there was no inferential statistics done hence the multivariable analysis part has been omitted.***

4. When comparing your findings to existing literature, you reference high-income countries well, while there are some references from sub-Saharan Africa but the manuscript lacks sufficient studies from sub-Saharan Africa or other LMICs. Including these would make your findings more regionally relevant.

***Answer:***

***We have exhausted existing literature for LMICs to the best of our knowledge, which is why we also included some literature for HICs in the discussion.***

5. The policy implications are strong, particularly the recommendation for geriatric-specific guidelines. To make it actionable, it would help to include examples of how guidelines from high-income countries could be adapted to Tanzania, which is currently missing.

***Answer:***

***We have highlighted examples of CPGs implementation in LMICs***

6. The abstract is concise and informative, but it would be more impactful if it included a key policy takeaway or actionable recommendation, which is absent right now.

***Answer:***

***We have included a key intervention based on our findings as part of the next steps of this project, in the conclusion section.***

7. The introduction sets the stage well, but it doesn’t sharply highlight the specific gaps in Tanzanian healthcare policies for older adults. A clearer framing of these gaps could strengthen the narrative.

***Answer:***

***We have highlighted the lack of standard geriatric protocols in the current clinical practice in Tanzania.***

8. The results section is comprehensive, but the inclusion of numerous detailed tables can overwhelm readers. Moving less critical tables to supplementary materials could help streamline the presentation.

***Answer:***

***We have only 4 descriptive tables showing comparison of different characteristics and outcomes between older and younger patients. They all present key information which might of interest to the readers. We have revised the tables for clarity.***

9. The tables and figures are clear, but the text often repeats data already presented visually. Summarizing key takeaways instead would make the narrative more concise and engaging.

***Answer:***

***The text has been revised to summarize key results from the tables.***

10. The conclusion emphasizes the need for tailored guidelines, which is excellent. However, it would benefit from a stronger call to action, such as training programs or resource allocation, which is currently missing.

***Answer:***

***We have added the next steps following the evidence, which is the implementation of CPGs for geriatric care in our setting, including capacity building components. The CPG will also facilitate proper allocation of resources.***

11. The references are solid, but there’s a lack of studies from sub-Saharan Africa or LMICs. Adding these would enhance the contextual relevance of the manuscript.

***Answer:***

***We have exhausted existing literature from LMICs to the best of our knowledge.***

12. The authors should consider making the de-identified dataset used in the study available on a public platform, such as GitHub, Dryad, or Figshare. This would enhance the transparency, reproducibility, and impact of the research, allowing other researchers to build upon these findings. Highly recommend doing so... This would also comply with the PLOS Data Policy which requires “PLOS journals require authors to make all data necessary to replicate their study’s findings publicly available without restriction at the time of publication.”

***Answer***

***We are making efforts to make our trauma registry data FAIR and in line with the FAIR principles. However, the data is currently available upon request to authors due to the local policies and regulations in the country concerning data sharing and ownership.***

**Reviewer #2:**

In this research article, the authors compared demographic and clinical features of two groups of adults traumatized patients (18-60 years and >60 years of age) treated between 2020-2024 at a trauma hospital in Tanzania using prospectively collected registry data. Primary outcomes included length of hospital stay and in-hospital mortality. They found significant differences between the two groups, which may be useful to optimize care pathways of the older population.

In my view, the article can be of interest for the readership of Plos Global Health but requires some adjustments:

**Abstract:**

- An analysis of in-hospital complications is anticipated in the 1st paragraph, but this is not described in the results section.

What is NCD?

***Answer:***

***We revised the section to precisely in-hospital outcomes which we analyzed in the study.***

**Introduction:**

- The main research question is well summarized and overall well written, There are some un-necessary repetitions: “As of late, people aged 60 and above make up ~6% of the population in Tanzania and ~10% in the Kilimanjaro region” AND “In the Kilimanjaro region people aged 60 and above make up (10.4%) of the population, which…”

***Answer:***

***We have revised the introduction and removed repetitions.***

**Methods**

- What type of registry was used? Some details about it?

***Answer:***

***We have added details about the KCMC Trauma registry in the study design/setting section.***

- No need to repeat two times the time interval

***Answer:***

***We have revised the methods and removed repetitions***

- This study used ALL trauma patients who presented, should be corrected to ALL ADULTS…

***Answer:***

***This section was revised as advised***

- "We defined in-hospital death as the death of a patient at any time after

admission to the hospital and as a reason for discharge”: what does this mean?

***Answer:***

***We revised this section for clarity.***

- It should be mentioned that also the site of injury was analyzed and not only the type of injury.

***Answer:***

***We have revised the variables section to include all the variables analyzed***

- Results, discussion, and conclusions:

The conclusions are supported by the results and adequate. Limitations are clearly stated.

**General comments:**

- A revision of the English language is necessary, including less verbosity in sentences. The explanation of abbreviations don’t need to be repeated along all the article: once described at the beginning, just use the abbreviation in all the article.

***Answer:***

***We have revised the writing, especially in the methods and results section. Also we have revised unnecessary repetitions.***
